# Issues in the Management of Acute Agitation: How Much Current Guidelines Consider Safety?

**DOI:** 10.3389/fpsyt.2013.00026

**Published:** 2013-05-07

**Authors:** Bruno Pacciardi, Mauro Mauri, Claudio Cargioli, Simone Belli, Biagio Cotugno, Luca Di Paolo, Stefano Pini

**Affiliations:** ^1^Psychiatry Division, Department of Psychiatry, Neurobiology, Pharmacology and Biotechnology, University of PisaPisa, Italy

**Keywords:** psychomotor agitation, rapid tranquilization, treatment guidelines, safety, tolerability, parenteral treatment, torsade de pointe, respiratory depression

## Abstract

Agitated behavior constitutes up to 10% of emergency psychiatric interventions. Pharmacological tranquilization is often used as a valid treatment for agitation but a strong evidence base does not underpin it. Available literature shows different recommendations, supported by research data, theoretical considerations, or clinical experience. Rapid tranquilization (RT) is mainly based on parenteral drug treatment and the few existing guidelines on this topic, when suggesting the use of first generation antipsychotics and benzodiazepines, include drugs with questionable tolerability profile such as chlorpromazine, haloperidol, midazolam, and lorazepam. In order to systematically evaluate safety concerns related to the adoption of such guidelines, we reviewed them independently from principal diagnosis while examining tolerability data for suggested treatments. There is a growing evidence about safety profile of second generation antipsychotics for RT but further controlled studies providing definitive data in this area are urgently needed.

## Introduction

Agitated behavior constitutes up to 10% of emergency psychiatric interventions (Tardiff and Sweillam, 1982). The overall prevalence of agitation in patients with schizophrenia or mood disorder is about 11–13%, with even higher rates among individuals with alcoholism (25%) or substance misuse (35%) (Swanson et al., 1990), dementia (24–45%), anxiety disorders (20–30%).

Agitation has rarely been the primary focus of both phenomenological classification and therapeutic intervention. In addition the study of agitation in mental illness has been complicated by a host of often imprecise or conflicting definitions (Mintzer, 2006). Available evidences indicate an extreme variability in the management of agitation cases in emergency settings (Bourdinaud and Pochard, 2003; Chan et al., 2011). Notwithstanding, rapid and effective pharmacological treatment is often required to ensure the safety of patients, and caregivers.

Allen (2000) defines agitation as “a temporary disruption of the typical physician–patient collaboration, which interferes with assessment and treatment, during a period when immediate assessment and treatment are needed.” Such therapeutic need can be answered by rapid tranquilization (RT), an approach consisting in the assertive use of medication to quickly calm severely agitated patient, decrease dangerous behaviors, and allow treatment of the underlying condition (Dubin and Feld, 1989; Karagianis et al., 2001; Mantovani et al., 2010). Therefore, the ultimate goal of RT is not treating the underlying disorder, but rather reestablishing a normal physician–patient relation.

Agitation comprises verbal and physical, aggressive and non-aggressive components. Its hallmarks are motor restlessness, heightened responsivity to internal and external stimuli, irritability, inappropriate or purposeless verbal or motor activity, in addition with vegetative signs and an unstable course (Lindenmayer, 2000). As a consequence, RT may be conceptualized as initial intervention that cuts across different disorders, being focused more on acute symptoms than specific diagnosis (De Fruyt and Demyttenaere, 2004). Behavioral manifestations of agitation are important therapeutic targets of drug treatment. However, specific endpoints have not been well defined yet. For example “sleep” is sometimes considered as a valid treatment target for agitation reduction, while “tranquillization” (a calming process different from total sleep induction) has been identified by many clinicians as the ideal therapeutic endpoint for the treatment of agitation (Battaglia et al., 2003).

Pharmacological management of agitation in individuals with psychiatric disorders is under-researched (Alexander et al., 2004). In particular, RT has not a strong evidence base, with recommendations being partly based on research data, partly on theoretical considerations and partly on clinical experience (Ferrier, 2006). The few available guidelines on this topic describe pharmacological and behavioral interventions of RT in emergency settings regardless of further diagnostic process.

The need for drug delivery in uncooperative patients favors the use of intramuscular preparations. Intramuscular treatment options include BDZ, FGA, and SGA. Each of these medications offers a unique pharmacological profile that must be considered when treating agitated patients, who may be unwilling or unable to adequately communicate their medical status and concomitant medications (Zimbroff, 2008).

Intramuscular injections of FGA and BDZ, given alone or in combination, have been the treatment of choice over the past few decades, with haloperidol and lorazepam being among the most widely used agents for agitation (Battaglia, 2005).

Most recent approved guidelines (see Table 1 for details) on this topic recommend the use of SGA, FGA, and BDZ. They also include recommendations for specific compounds such as haloperidol, chlorpromazine, diazepam, lorazepam, and midazolam. However, these latter five drugs, according to most recent evidences, have questionable tolerability profiles. In particular, there are growing evidences indicating that haloperidol is involved in a potentially lethal cardiac arrhythmias named “torsade de pointe.” This finding led European drug authorities to issue a black box warning. After that a prolongation of QTc interval in ECG of some individuals treated with haloperidol was related with fatal torsades de pointes the use of this drug became strictly regulated, especially in its parenteral formulation (Hunt and Stern, 1995; Jackson et al., 1997; Tisdale et al., 2001; Hassaballa and Balk, 2003; Meyer-Massetti et al., 2010).

**Table 1:** **Guidelines – recommended drugs and safety considerations**.

Guidelines	Reference	Diagnosis	Recommended drugs	Missing safety considerations	Reported safety and efficacy considerations
Rationale and guidelines for inpatient treatment of acute psychosis	Feifel (2000)	Acute psychosis	SGAs: first-line treatment FGAs: haloperidol recognized as gold standard in comparative clinical studies assessing SGAs efficacy BDZs: lorazepam or comparable BDZ to control breakthrough agitation control or as augmentation therapy	No mention of arrhythmia / hypotension with FGAs No mention of respiratory depression with BDZs or preventive requirement of flumazenil availability	
The World Federation of Societies of Biological Psychiatry (WFSBP) Guidelines for the biological treatment of bipolar disorders, Part II: treatment of mania – 2003	Grunze et al. (2003)	Acute mania	SGAs: first-line treatment* FGAs: restricted to very severe/violent cases of mania	No mention of arrhythmia Quote: “for a variety of antipsychotics prolongation of the QT interval may constitute a minor additional problem.”	*Efficacy of SGAs in acute mania still needs to be established in comparison with FGAs
The Expert Consensus Guideline Series: treatment of behavioral emergencies. A postgraduate medicine special report – May 2001 (American Association of Psychiatric Emergency)	Allen et al. (2001)	Schizophrenia, mania, psychotic depression (provisional)	FGAs: first-line treatment (administration of high potency AP + BDZ) SGAs: where possible orally, associated with BDZ in mania BDZs: first-line in intoxication, and non-alcohol substance abuse FGAs and BDZs: second-line option in intoxication, substance, and alcohol abuse	No mention of arrhythmia / hypotension with FGAs. Not any adjunctive safety consideration for BDZs in pts with alcohol abuse Parenteral administration in uncooperative patients with provisional diagnosis imply partial assessment of medical risk profile	
The expert consensus guideline series. treatment of behavioral emergencies 2005	Allen et al. (2005)	Schizophrenia, mania, psychotic depression (provisional)	BDZs: first-line treatment where no data available SGAs: not one emerges as alternative to haloperidol FGAs: haloperidol when no data available or medical comorbidities (chlorpromazine as third line)	Haloperidol mono-therapy suggested only in the medically compromised patient without any specification about cardiovascular functioning	Safety consideration about possible hypotension with chlorpromazine
The World Federation of Societies of Biological Psychiatry (WFSBP) – acute treatment of schizophrenia	Falkai et al. (2005)	Acute treatment of schizophrenia	SGAs: first-line treatment FGAs: low dosage as first-line alternative to SGA’s BDZs: “regular and liberal doses of benzodiazepines” as adjunctive treatment	No mention of BDZ-related respiratory depression risk No mention of flumazenil availability requirement	Mention of Qtc prolongation risk with high-dose intravenous Haloperidol (Al-Khatib et al., 2003)
Scottish Intercollegiate Guidelines Network	Ebmeier et al. (2005)	Mania	FGAs: (including chlorpromazine and haloperidol) among emergency first-line drugs BDZs: (Lorazepam) add-on where sedation is a priority. SGAs: lesser evidence than FGA’s	No mention of cardiovascular risk with i.m. chlorpromazine and e.v. haloperidol	FGA’s used as “gold standard” (including chlorpromazine and haloperidol) to validate effectiveness of new drugs Lack of placebo-controlled data on FGA’s efficacy in acute mania
Canadian Network for Mood and Anxiety Treatments	Yatham et al. (2005)	Acute mania	SGAs: first-line medication in association with mood stabilizers FGAs: haloperidol or chlorpromazine third-line treatment in association with mood stabilizers	No mention of FGA-related cardiovascular risk or hypotension	
NICE Guidelines: the management of bipolar disorder in adults, children, and adolescents in primary and secondary care	Ferrier (2006)	Acute mania	SGAs: first-line treatment (mono-therapy or add-on with mood stabilizers) FGAs: haloperidol as single agent for RT therapy BDZs: lorazepam indicated as single agent in RT or add-on with SGAs SGAs and FGAs as a group for marked behavioral disturbances or psychotic symptoms	No mention of arrhythmia cardiovascular risk with IM haloperidol No mention of flumazenil availability requirement	Diazepam and chlorpromazine are not recommended for routine use in bipolar disorder “Olanzapine and BDZ should not be given intramuscularly within 1 h of each other” RT with olanzapine, lorazepam, or haloperidol, wherever possible as a single agent Mention of respiratory depression with BDZ
					Mention of QTc prolongation with withdrawn FGA’s and mention of hypotension risk with chlorpromazine
					Generic caution with newer agents (SGA’s)
A review of agitation in mental illness: treatment guidelines and current therapies	Marder (2006)	Psychomotor agitation	SGAs: first-line treatment BDZs: Lorazepam as first-line, although other BDZ recommended (diazepam, midazolam) Midazolam found to be superior to haloperidol on motor agitation control	Controversial statements about cardiovascular side effects of FGA’s and QTc prolongation-related fatalities Haloperidol considered to have relatively low risk for QTc prolongation/torsade de pointe	Close patient monitoring suggested Quinidine-like cardiac effects of all FGA’s and sudden death cases reported
				BDZ registration issues (midazolam)	Caution suggested with midazolam use in patients with pulmonary reserve limiting conditions Mention of BDZ-related respiratory depression risk
The World Federation of Societies of Biological Psychiatry (WFSBP) update 2009 on the treatment of acute mania	Grunze et al. (2009)	Acute mania	SGAs: aripiprazole, ziprasidone among first-line treatment (add-on with mood stabilizers) FGAs: efficacy of haloperidol and chlorpromazine across subtypes of mania, including emergency treatment or non-responders	No mention of haloperidol: related arrythmia risk No mention of chlorpromazine related cardiovascular risk or hypotension BDZs: lack of placebo-controlled studies	Paucity of placebo-controlled studies about chlorpromazine use in acute mania mentioned
			BDZs: lorazepam only as add-on	
2009 Maudsley Guidelines for the treatment of acutely disturbed or violent behavior	Taylor et al. (2009)	Acutely disturbed violent behavior	SGAs: treatment of choice for antipsychotic naïve patients FGAs: haloperidol in antipsychotic naïve patients, prometazine for patients already under AP treatment BDZs: lorazepam for patients already taking antipsychotic treatment IM treatment is specifically considered a second-line approach between oral and IV treatment	BDZ registration Issues (midazolam) No mention of potential toxic/organic causes of agitated/violent that may require additional safety consideration	Explicit safety recommendations: concomitant use of antipsychotics to be avoided due to QT prolongation risk. Haloperidol use considerations Mono-therapy mandatory Pre-treatment ECG required–haloperidol should be the last drug considered BDZ: Shouldn’t be administered in combination with olanzapine
					Flumazenil availability mentioned
					During RT vital signs should be monitored

Diazepam and midazolam administration bear a strong respiratory function depression risk, especially in patients with conditions limiting respiratory functions, or BDZ metabolism (hepatic impairment or alcohol abuse) (Denaut et al., 1974/1975; Jedeikin et al., 1985; Altose and Hudgel, 1986; Peppers, 1996; Nordt and Clark, 1997; Brice et al., 2003; Arcangeli et al., 2005; Boomsma et al., 2006). Lorazepam infusion has also been involved in cases of renal glycol toxicity, especially in patients with substance use (i.e., cocaine) (Cawley, 2001; Arcangeli et al., 2005; Wilson et al., 2005; Zar et al., 2007; Riker and Fraser, 2009).

Another example of the discrepancies between what claimed in most recent guidelines and what reported in the literature is the case of chlorpromazine. Several articles, in fact, report an association of this compound with a high risk of cardiovascular complications, in particular severe hypotension, given the high doses required for RT (Fruensgaard, 1978; Hoehns et al., 2001; Wilson et al., 2005; Ahmed et al., 2010; Muench and Hamer, 2010). From this perspective, it is difficult to understand why guidelines still recommend parenteral use of BDZ, chlorpromazine, and haloperidol for RT despite these important safety concerns.

The purpose of this work was to assess a series of guidelines pharmacological recommendations about RT specifically focusing on safety issues. We tried to evaluate safety considerations with particular reference to the use of haloperidol, chlorpromazine, diazepam, lorazepam, and midazolam.

## Method

In this paper we reviewed guidelines published from 2000 to 2010 including recommendations about pharmacological treatment of agitation (see Allen’s definition 3) in psychiatric emergency settings.

With this purpose, a computerized search for guidelines was conducted using “PUBMED.” Medical subject heading terms used for our search were: “agitation,” “acute agitation,” “psychomotor agitation,” “behavioral agitation,” and each of these terms was combined (by Boolean operator AND), combined with each of the following terms, as medical subject headings: “rapid tranquillization,” “tranquillization,” “treatment,” “drug treatment,” “pharmacological treatment,” “management.” Data resulting from computer searches were integrated with available published guidelines.

The search was initially restricted to guidelines as publication type and keyword. No randomized controlled studies or clinical studies were considered eligible for this particular data selection. Guidelines were selected and assessed as for drug suggestions and safety considerations. We focused exclusively on safety issues that may endanger the patient undergoing parenteral treatment in emergency setting.

We performed a new computerized search for articles pertaining the safety profile of some of the compounds suggested in guidelines for RT of agitated patients (i.e., haloperidol, chlorpromazine, diazepam, lorazepam, midazolam, olanzapine, aripiprazole, and ziprasidone). The choice of drugs included in our new search were based on the following criteria: their belonging to FGAs, BDZ, or SGAs pharmacologic class, being widely used in emergency settings, being available in intramuscular formulations. This computerized search for articles was conducted using “PUBMED” by searching for medical heading terms: “tolerability,” “adverse effect,” “adverse reaction,” “fatality,” “side effect,” “risk,” combining these terms with the Boolean operator “AND,” and then combining them with each of the above mentioned compounds. In this data selection, we extended our search to reviews and randomized controlled trials with no restriction to a specific medical condition.

After a revision of collected data pertaining the safety profile of all of the selected compounds we listed the main safety issues related to each of them, compared the results of our search, and then we tried to provide some considerations.

## Guidelines in Detail

In the “Rationale and guidelines for inpatient treatment of acute psychosis,” Feifel clearly suggested SGAs as first-line treatment, while pointing out that competitor-controlled studies of SGA efficacy still consider haloperidol as “…gold standard treatment…” Lorazepam (or comparable BDZ) was suggested as a possible augmentation therapy. No particular mention of arrhythmia or hypotension risk with FGAs was reported and neither was the risk of respiratory depression with high potency BDZ (Feifel, 2000). Grunze et al. (2003) reported that recommended SGAs dosages for schizophrenia may be too low for controlling agitation in severe mania and that the efficacy of SGAs in comparison with FGAs needs to be confirmed by further studies. The same authors recommend to restrict the prescription of parenteral high doses of FGAs to very severe or violent cases of mania. On the safety side, authors state that, for a variety of antipsychotics, prolongation of the QT interval may constitute a minor additional problem (Grunze et al., 2003).

A different approach to short-term management of agitation is adopted by Allen et al. (2001). These authors suggest to take into account provisional diagnosis (for instance, schizophrenia, mania, psychotic depression) in drug treatment intervention. Parenteral injection of a high potency FGA in association with a BDZ is indicated as the treatment of choice in non-collaborative patients. However oral BDZ and SGAs should be preferred in all other cases. As for the safety profile, we found no mention of respiratory depression risk, and the role of BDZ in alcohol intoxication is far from being clear: on one side there is lack of consensus among authors on BDZ use as a first-line treatment in such condition, on the other there is no clear distinction between alcohol withdrawal and intoxication where BDZ are indicated as second-line treatment (Allen et al., 2001). The same authors undertook a new survey in 2005. In this update, BDZ were suggested as first-line treatment for agitation when no patient data are available. No single SGA emerges as an alternative to haloperidol.

None of the new SGA parenteral formulations received as much support as traditional agents (i.m. BDZ, i.m. haloperidol) when no patient data are available, or the diagnosis includes medical comorbidity or state of intoxication. Among suggested FGAs, chlorpromazine received only third-line ratings. Safety issues report a warning about severe hypotension associated to chlorpromazine.

It seems noteworthy that haloperidol was suggested, in mono-therapy, “only in the medically compromised” patients and also in absence of previous patient information, regardless of specific medical condition that may further increase cardiovascular risk in such patients (Allen et al., 2005).

The Scottish Intercollegiate Network Guidelines for the treatment of acute mania (2005) suggested parenteral administration of antipsychotics (including haloperidol and chlorpromazine), proposing add-on therapy with BDZ, such as parenteral lorazepam, for emergency situations where sedation is a priority. Although the authors suggest the use of parenteral FGAs and BDZ to obtain sedation, they do not provide considerations about potential cardiovascular risk with administration of haloperidol or chlorpromazine and they do not mention respiratory depression risk related to BDZ use (Ebmeier et al., 2005).

In 2005 World Federation of Societies of Biological Psychiatry guidelines for acute treatment of schizophrenia the suggested first-line treatment consisted in SGAs (and, alternatively, low doses of FGAs), with adjunctive BDZ to relieve distress, insomnia, and behavioral disturbances secondary to acute psychosis. The authors suggest “regular and liberal doses of BDZ” without any mention of respiratory depression risk. Interestingly, these same guidelines mention the risk of QTc prolongation associated with high doses of intravenous haloperidol (Falkai et al., 2005).

A further indication of SGAs as first-line treatment for agitation can be found in 2005 Canadian guidelines for acute mania treatment, where olanzapine, risperidone, quetiapine, aripiprazole, ziprasidone are recommended as first-line treatment in association with lithium or divalproate. In these guidelines FGAs (haloperidol or chlorpromazine) are suggested as third-line treatment in association with mood stabilizers, without any consideration about the associated cardiovascular or hypotension risk (Yatham et al., 2005).

The National Clinical Practice Guidelines for acute mania treatment (Ferrier, 2006) recommend antipsychotic medication only when the clinical presentation includes psychotic symptoms or marked behavioral disturbances. In these cases, SGAs (aripiprazole, olanzapine, quetiapine, risperidone, ziprasidone) in association with mood stabilizers (valproate or lithium) are suggested as first-line oral treatment; IM administration of lorazepam, olanzapine, or haloperidol is recommended when RT is needed. Regarding safety issues, the authors mention respiratory depression, ataxia, sedation, and falls during BDZ treatment. These guidelines recommend prompt availability of flumazenil and the necessity of avoiding concomitant use IM olanzapine and BDZ within an hour. Beside these safety recommendations, though, there was no explicit mention of cardiovascular risk of arrhythmia with IM haloperidol, while risk of QTc prolongation was reported for droperidol and thioridazine (both withdrawn from market). Authors specified that there was no RCT evidence for the use of BDZ in acute mania. The same authors also suggested to avoid chlorpromazine because of hypotension risk. As for SGAs they were reported to cause less EPS than FGAs but the authors advise to be cautious with new agents (Ferrier, 2006).

In a review paper published in the same year, Marder (2006) assessed psychomotor agitation across different mental illnesses, with a further proposal of treatment guidelines: once again SGAs are recommended as first-line treatment, with haloperidol being considered among available FGAs.

As for safety issues, cardiovascular risk related with antipsychotics is reported with at least controversial statements. On one side it was reported that all FGAs have the potential to increase the risk of cardiac arrhythmia with reports of sudden death occurring during RT. On the other hand, the authors pointed out that, in these deaths, the role played by antipsychotics remains unclear since most fatalities had multiple probable causative factors.

As to BDZ, IM lorazepam remained the most popular one for use in agitation. It is interesting to note that midazolam (regardless of registration issues) was found superior to haloperidol on motor agitation control with respect to diazepam and chlordiazepoxide. As for the safety of these drugs, BDZ were not recommended in patients with conditions that limit pulmonary reserve given the risk of inducing respiratory depression, ataxia, excessive sedation. The authors concluded that close monitoring of patients receiving pharmacotherapy for acute agitation was imperative (Marder, 2006).

In a 2009 update of WFSBP guidelines for treatment of acute mania SGAs (aripiprazole, risperidone, and ziprasidone) plus mood stabilizers (valproate) are suggested as first-line treatment. Among FGAs, haloperidol efficacy is reported across all subtypes of mania and its use suggested in emergency treatment of severe mania or in non-responders. For the same conditions treatment with chlorpromazine is also suggested, pointing out that only one small placebo-controlled study report its efficacy in acute mania. No cardiovascular risk related to haloperidol or hypotension risk related to chlorpromazine is mentioned (Grunze et al., 2009).

In 2009 Maudsley Prescribing Guidelines section on the treatment of acutely disturbed or violent behavior, the authors suggest lorazepam or promethazine (oral or IM) for patients taking antipsychotic treatment, while SGAs (olanzapine, aripiprazole, IM or oral, oral risperidone) are considered treatment of choice for antipsychotic naïve patients. As for safety issues, concomitant use of two or more antipsychotics should generally be avoided due to greater QT prolongation risk. For the same reason, antipsychotic mono-therapy is considered mandatory in patients receiving haloperidol. During haloperidol therapy pre-treatment ECG is required and haloperidol should be the last drug to be taken into account, according to authors indications. BDZ should not be administered in combination with IM olanzapine. The authors also specify that, during RT, temperature, pulse, blood pressure, and respiratory rate should be monitored (Taylor et al., 2009).

## Discussion

Some limitations of this work must be acknowledged. First, we have included in our revision the most important and widely accepted guidelines, however it is possible that we missed to revise some of the many available contributions on this topic. Second, agitation is a heterogeneous concept crossing multiple diagnostic areas in psychiatry. Therefore, one could argue that specific interventions should vary according to principal psychiatric diagnosis. However, we explicitly focused on treatment recommendation for controlling acute symptoms and behavioral disturbances related to agitation independently of underlying psychiatric condition. Even in emergency setting, therapeutic choices should be guided by an assessment of risk/benefit ratio: therefore, another limitation of our study could be acknowledged in our reviewing only the risk profiles of guideline-recommended interventions.

The aim of this work was to review guidelines suggestions about RT of acutely agitated patients in a real world setting where provisional diagnosis are common, severe agitation requires parenteral treatment, and rapid decisions are needed. With these conditions in mind, we tried to focus on safety issues related to the application of existing guidelines in order to check if they could help clinicians to lessen the inherent risk of weighing efficacy versus safety in a very short time. In such a setting, when possible, blood samples, urinary drug screening, complete physical examination should always be performed along with a treatment history in order to assess concomitant medical conditions or substance abuse.

Given the necessity for immediate treatment in patients suffering from agitation there is a warranted clinical need for very rapid and well tolerated pharmacological intervention. As a result treatment suggestions for agitated patients rely heavily on administration of intramuscular antipsychotics or BDZ. Aside for drugs’ efficacy profiles, clinicians should be aware of the risks related with the high dosage required.

Taken as a whole, drug treatment guidelines of the agitated psychiatric patient recommend the use of SGAs as first-line compounds. Among feasible options, the use of FGAs and BDZ is still recommended (or at least considered) for RT despite the increasing amount of worrisome data about their safety, with little or no mention of the now well established risk of fatalities related to their use. On the other hand, we found extensive considerations regarding SGA-associated safety; therefore an a-critical application of suggestions reported in available guidelines may imply significant risks for the agitated patients undergoing RT.

The major safety concerns about treatment with SGAs include metabolic effects (weight gain, hyperglycemia, and hyperlipidemia). Notwithstanding, these aspects are not particularly relevant within the framework of acute agitation management.

More complex is the issue of potential arrhythmogenic effects of antipsychotics.

There are anecdotal reports of QTc prolongation with SGAs. Furthermore, in a recent study examining cardiovascular effects after SGA medication overdose, no reports of ventricular dysrhythmias (including torsade de pointes) could be clearly attributed to atypical antipsychotic acute administration (Tan et al., 2009).

Few reports in small samples of patients undergoing intramuscular olanzapine – intramuscular BDZ combination treatment for agitation seem to indicate lower oxygen saturations especially in patients with alcohol abuse (Wilson et al., 2012a,b). Furthermore, we have found limitations in the use of such a combination in the guidelines examined. There are, therefore, sufficient data to limit the use of this association in agitated patient. Further studies are needed to clarify definitively this issue.

As a final remark, we would like to summarize a few basic safety considerations (Tables 2 and 3), together with some pharmacokinetic parameters (Table 4), that we find useful to keep an evidence based security profile in treatment planning.

**Table 2:** **Safety procedures (required or suggested) to reduce the risk of severe adverse effects during IM treatment of agitation**.

Im drug	Main risks	Reference	Compulsory safety procedure	Useful safety procedures
Haloperidol	Torsade de pointe	Meyer-Massetti et al. (2010)	Pre-treatment ECG required	Post-treatment ECG
		Tisdale et al. (2001)	No antipsychotic combination	Electrolyte balance assessment
		Hassaballa and Balk (2003) Hassaballa and Balk (2003) Jackson et al. (1997)	No familiar, preceding or ongoing cardiovascular disorder No concomitant drug affecting QTc	Tox-screen Neurological assessment
			No concomitant General Medical Condition affecting QTc	
Chlorpromazine	Severe hypotension	Ahmed et al. (2010)	Pressure monitoring	Test dose before full dose
		Muench and Hamer (2010)		Pre-treatment ECG useful
				Electrolyte balance assessment
Lorazepam	Respiratory depression (lower)	Gillies et al. (2005) Riker and Fraser (2009)	No drugs or GMC affecting respiratory function	Blood test Check for acidosis / anion gap
	Propylene glycol toxicity	Cawley (2001)	Flumazenil available	Check for alcohol intoxication
		Wilson et al. (2000)		
		Zar et al. (2007)		
		Arcangeli et al. (2005)	
Diazepam	Respiratory depression Propylene glycol toxicity	Denaut et al. (1974/1975) Boomsma et al. (2006) Arcangeli et al. (2005)	No drugs or General Medical Condition affecting respiratory function	Blood test useful Check for acidosis / anion gap Check for alcohol intoxication
		Brice et al. (2003)	Flumazenil available	
		Peppers (1996)		
		Zar et al. (2007)	
Midazolam	Respiratory depression (high)	Nordt and Clark (1997) Boomsma et al. (2006) Huf et al. (2005)	No drugs or general medical condition affecting respiratory function	Informed consent (off label) Blood test useful Check for alcohol intoxication
		Spain et al. (2008)	Flumazenil available	
Olanzapine	Cardiorespiratory depression in combination with BDZs	Wilson et al. (2012a) Wilson et al. (2012b)	Avoid BDZs combination	None
Ziprasidone	QTc prolongation (controversial)	None	None	Pre-treatment ECG
Aripiprazole	None	None	None	None

**Table 3:** **Conditions and safety considerations that should preclude specific drug choice during IM treatment of agitation in emergency settings due to fatality risk**.

Condition	Safety consideration	Drug to avoid
Alcohol abuse/intoxication	Possible respiratory distress	BDZs
Opioid intoxication	Possible respiratory distress	BDZs
General medical condition affecting resp. funct	Possible respiratory distress	BDZs
General medical condition affecting card. funct	Cardiovascular risk	FGAs
No informations	Possible cardiovascular/Qtc affect. cond.	Haloperidol
Impossible assessment of resp. function	Possible respiratory distress	BDZs
Delirium	Possible worsening	BDZs
Stimulants intoxication	Cardiovascular risk	FGAs
Impaired renal function/cocaine	Propylene glycol toxicity	lorazepam, diazepam
BDZs pre-treatment	Cardiorespiratory depression	Olanzapine

**Table 4:** Basic pharmacokinetic profile of discussed IM drugs (Goodman et al., 2006; *Smith et al., 1981).

Drug	Plasma peak time	Elimination pathway	Half-life (average)	Active metabolites
Haloperidol	20′	Hepatic	24 h	Hydroxy-haloperidol
Chlorpromazine	15’	Hepatic	15–30 h	7-Hydroxychlorpromazine chlorpromazine N-oxide (possibile)
Lorazepam	2–6 h	Hepatic	10–20 h	None
Diazepam	1, 5 h	Hepatic, renal	4–6 h	Desmethyldiazepam, oxazepam
Midazolam*	24′	Hepatic	1–3 h	None
Olanzapine	5-8 h	Hepatic	21–54 h	None
Ziprasidone	6, 6 h	Hepatic	6–8 h	*S*-methyldihydroziprasidone
Aripiprazole	3–5 h	Hepatic	75–146 h	Dehydroaripiprazole

Randomized case control studies are urgently needed in order to solve the questions about safety of patients receiving pharmacological treatment in psychiatric emergency settings.

## Conflict of Interest Statement

Professor Mauro Mauri declares past financial relationship as a speaker for potentially interested parties as Janssen, Lilly, Lundbeck, Servier. No other author declares financial, commercial or relevant relationship representing a potential conflict of interest.
